# The rates of hospital admissions and return visits to a rapidly growing pediatric emergency department as measures of quality of care

**DOI:** 10.1186/s13584-020-00397-y

**Published:** 2020-08-12

**Authors:** Amit Keret, Yakir Shir, Shepard Schwartz, Elihay Berliner, Mattityahu Erlichman, Giora Weiser

**Affiliations:** 1 Hebrew university medical school, Jerusalem, Israel; 2 Pediatric emergency department, Shaare Zedek Medical Center, 12 Bait st, Jerusalem, Israel

**Keywords:** Return visits, Pediatric emergency department, Admissions, Patient education

## Abstract

**Background:**

Return visits to the emergency department are viewed as a quality measure of patient management. Avoiding unnecessary admissions to the ward can potentially cause an increase in return visits, thus effecting quality assessment.

**Methods:**

After implementing an educational process the relationship between admissions and return visits was assessed over time at a rapidly growing pediatric emergency department.

**Results:**

There was a 264% increase in visits from 2004 to 2017. In the study period admission rates declined from 25 to 14%. This was achieved without a rise in return visits and with a stable percentage of admissions from return visits.

**Conclusions:**

Interventions aimed at decreasing unnecessary admissions do not lead to increased return visits and return visit admissions.

## Introduction

A pediatric emergency department (PED) serves as a health care facility for children who require emergent or urgent evaluation and possibly intervention (procedures or treatments) not available in the outpatient setting. The range of medical, surgical, orthopedic, and other conditions which bring children to the PED is wide, requiring the medical staff to effectively diagnose and treat a diverse array of emergencies, some of which may be imminently life-threatening. The PED therefore must continuously examine quality of care it provides. Quality assurance (QA) of a PED is difficult to assess, as it is multifactorial [1, 2] (hospital, physician and patient related factors) [3].

Two outcome measures commonly assessed are hospitalization rates and return PED visits (RV) [4, 5]. Inpatient hospitalization of a child may pose a financial burden on the family and as well carrying a risk of iatrogenic complications. These include medical errors, nosocomial infections and psychological stress, all of which impact patients and their families. However, unnecessary hospital admission, defined as hospitalization which offers no benefit compared to being treated as an outpatient, is a common phenomenon, found in up to 30% of hospitalized children [6].

A RV can indicate treatment failure or lack of patient education. Commonly, RVs are classified as early (within 72 h) or late (7–30 days) [6, 7]. The RV rate reported in most studies is approximately 5% but may be as high as 14%. In Israeli hospitals, the RV rates in the years 2011–2017 ranged from 4 to 7% in the 0–15 years of age groups. There is a mild yet constant decline in RVs over the years. RV rates are an important outcome measure, though it is unclear to what degree they reflect quality of care [8–12].

The PED of Shaare Zedek Medical Center (SZMC) in Jerusalem has grown rapidly over the last decade. Our new expanded facility opened in 2008. In 2012 the age limit of accepted patients was changed from 15 to 18 years. This, as well as other possible factors, led to a significant increase in the number of PED visits, from approximately 12,000 in 2008 to approximately 25,000 in 2015. The Jerusalem area population is a fast-growing population with an increase of close to 150,000 residents from 2008 to 2016. http://www.health.gov.il/PublicationsFiles/emergency_2013.pdf. This is an estimated 3% growth of the pediatric population.

In an attempt to avoid unnecessary hospitalization admissions, a systematic measures-based intervention was implemented:

- A senior attending physician in the PED was consulted for all cases for which hospitalization was recommended by a pediatric resident. The decision was ultimately made by the senior attending physician. In the absence of an attending, either a decision was made following a phone consult or the patient was seen when the attending arrived (This usually regards night hours). Children whose acute illness required only prolonged observation for up to 24 h were cared for in the PED instead of the inpatient ward (OA).
- A concerted effort was made to improve patient and family education. This was accomplished having the discharging physician detail the discharge recommendations as well as handing out specific hand-out notes on different common conditions.
- Improving the lines of communication with the patient’s primary physician. This was achieved by both contacting the primary care physician when available as well as having the PED attending available for phone contact when needed.

Improved patient management in the PED should result in a reduction in the rate of inappropriate hospitalization without a concomitant rise in RVs.

## Methods

A retrospective review via a computerized database of all patients treated in the PED of SZMC from January 2004 through December 2017 was performed. Data collected for each patient visit included age, gender, date of PED visit, season (categorized as winter [October–April] and non-winter), and final disposition, categorized as either discharge, OA or inpatient hospitalization.

Data was compared between 3 time periods: 2004–2007, before the opening of the new PED facility; 2008–2012, the first years of the new, expanded PED facility, and 2012–2017, the first years during which the PED treated all patients up to 18 years of age and all cases of minor trauma.

The primary outcome measure was the rate of hospitalizations during each of the 3 time periods. Secondary outcome measures included the percentage of RVs, percentage of RVs hospitalized and changes in OAs during each of the 3 time periods.

Data analysis was performed using Excel (Microsoft Corporation) and SPSS (IBM corporation). The data was assessed using the Chi-square tests to compare proportions of primary and secondary outcomes between various subgroups. *P* <  0.05 was considered statistically significant.

## Results

**Fig. 1:**
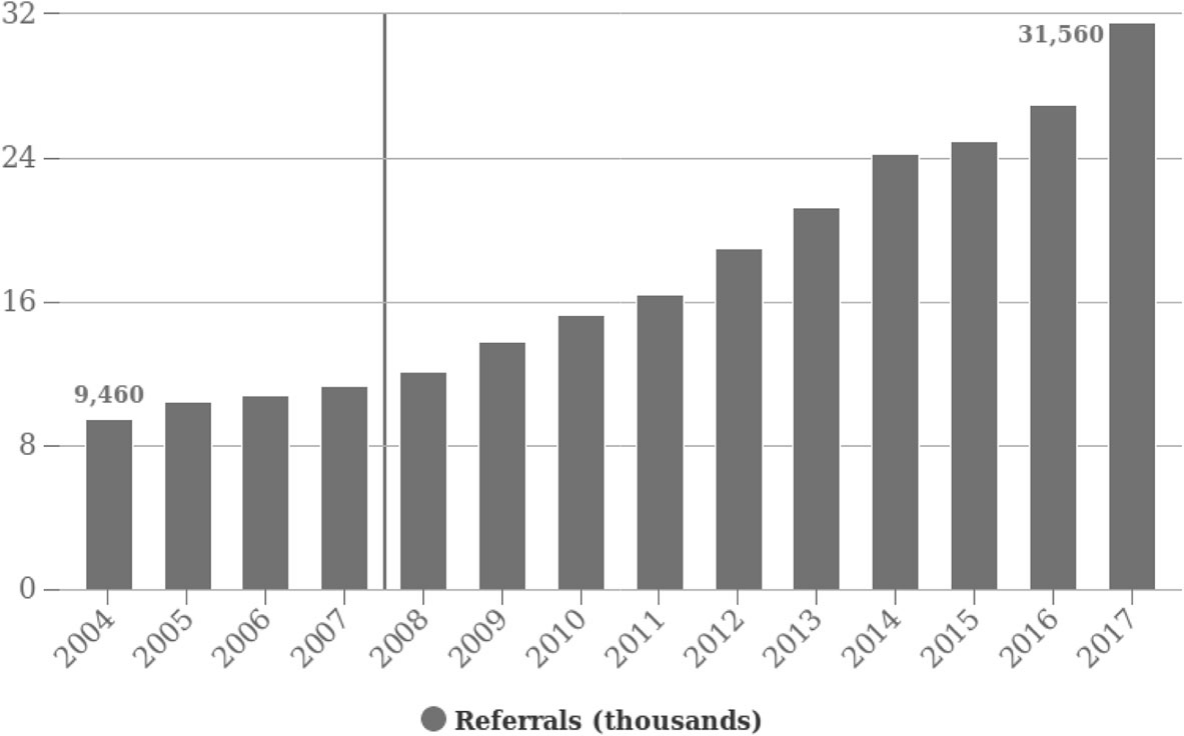
Change in admission over time

The number of PED visits rose by 264% from 9460 in 2004 to 31,560 in 2017 (Fig. 1).

During the years 2004–2007 the admission rate was approximately 25% (yearly range 25.1–27.0%). Starting in 2008 there was a gradual and consistent decline in admission rates which reached a plateau at 13–14% during the years 2012–2017. (Tables 1, 2).

**Table 1:** Overall changes in activity at SZMC PED 2004–2017

Year	Visits	Admission (%)	OAs (%)	RVs (%)	Admission of RVs (%)
**2004**	9460	27.0	11.1	3.3	38.8
**2005**	10,497	26.8	13.2	3.3	37.0
**2006**	10,780	25.3	14.3	3.1	35.9
**2007**	11,329	25.1	14.9	3.1	33.1
**2008**	12,119	20.7	15.4	3.8	27.2
**2009**	13,810	17.8	14.9	3.6	24.1
**2010**	15,298	16.7	14.6	3.8	28.3
**2011**	16,472	16.5	13.9	3.7	24.9
**2012**	18,961	15.0	12.1	3.1	21.7
**2013**	21,259	14.0	10.4	3.1	22.3
**2014**	24,270	13.5	9.9	3.2	22.1
**2015**	24,975	13.6	10.2	3.1	24.5
**2016**	26,964	13.7	7.5	3.2	23.8
**2017**	31,560	13.9	5.5	3.2	26.3

**Table 2:** Outcome measures subsequent to phased changes in PED patient management

	2004–2007	2008–2011	2012–2017	***P*** value
**Admission rate (%)**	26.1	17.9	14.0	< 0.001
**OAs rate (%)**	13.4	14.7	9.3	< 0.001
**RVs (%)**	3.2	3.7	3.2	0.005
**Admission rate from RVs (%)**	36.2	26.1	23.5	< 0.001

**Fig. 2:**
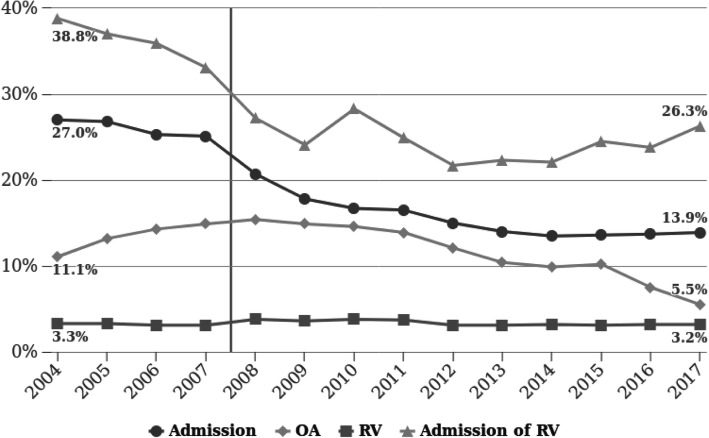
Changes in admissions and RVs 2004–2017

Compared to the years 2004–2007, admission rates following return visits, during the years 2008–2017 were significantly lower. There was also a small but significant decline in OAs between these two time periods, without a significant change in the rate of RVs. (Fig. 2). There was a small increase in RVs and OAs during the 2008–2011 period which was corrected in later years.

Comparison of outcome measures between winter and non-winter months showed no significant differences. Younger age was associated with a higher rate of hospital admission and RVs. The only outcome measure to differ between genders was the rate of OAs. (Table 3).

**Table 3:** Outcome measure analysis by age, gender and season

	Admission (%)	OAs rate (%)	RVs rate (%)	Admission from RVs (%)
Outcome Measures by age (yr)				
**0**	24.8	11.7	4.1	33.2
**1–4**	14.5	12.4	3.1	26.1
**5–16**	17.8	11.7	2.9	25.0
***P*** **value**	**< 0.001**	0373	**0.004**	0.154
Outcome measures by gender				
**Male**	18.4	11.4	3.4	27.2
**Female**	18.8	12.8	3.3	29.8
***P*** **value**	0.528	**0.007**	0.623	0.574

## Discussion

The findings of this retrospective study suggest that effective measures taken by the PED staff may prevent unnecessary hospital admissions. Since the rate of RVs did not increase in the time period during which there was a reduction in admission rate, the admission rate prior to the institution of the various measures was likely unnecessarily high. The reduction in admission rates without a concomitant rise in RV rates was achieved through a structured program implemented in the PED. Therefore, our findings also suggest that the combined analysis of hospital admission rates and RV rates provides more accurate quality of care information than the examination of each one independently.

The short-lived rise in OAs and RVs during the 2008–2011 period may be a result of the opening of a new PED facility and the need to standardize the patient flow and management.

Our findings of a higher rate of admissions and RVs among younger children as well as a lack of difference in admission and RV rates according to season are consistent with prior research [11]. Interestingly, females were more likely to incur an OA than males. The reason for this finding is unclear. This may have been a result of delays subsequent to gynecological consults. However, we were unable to determine the exact cause.

The 23.5–26.1% admission rate among RVs subsequent to 2008 are higher than the 16–19% rates reported in other studies. This may be due to our department’s adherence to the Israeli Health Ministry requirement to admit all RV patients who cannot be evaluated in the PED by an attending physician. Such physicians are present in our PED 16 h per day. The trends noted in our study should be viewed in the context of national ones during the study period. According to a report by the Israeli Ministry of Health http://www.health.gov.il/PublicationsFiles/emergency_2013.pdf, in 2008 the nationwide admission rate was 32 and 28% for children 1–4 and 4–15 years of age, respectively. In 2013, the national admission rates were 32% for infants under a year of age and 20% for children ages 1–4 years.

A review of the health ministry’s yearly reports during the study period https://www.health.gov.il/PublicationsFiles/emergency_2014.pdf shows that the addition of the 16–17-year age groups do not alter our findings. The average visit rate for these age groups was approximately 194 per 1000 of the population (range 190–198) throughout all of the study period. The return rates were similarly unchanged and thus unlikely to have a significant impact on the findings of this study.

In addition to its retrospective design, this study has other limitations. Our RV rates did not account for the possibility of children presenting to neighboring hospitals following discharge from the SZMC PED. Although we have the details of admissions and RVs at other Jerusalem hospitals, there is no way to determine which admissions were following a visit to SZMC PED. However, throughout the study period, the admission and RV numbers at neighboring hospitals changed minimally and cannot account for the changes seen at our PED.

Another factor which may have influenced our results is the category of OAs [13].

Our admission rate is based on the notion that observed patients remain under the PED care whereas admitted patients are treated on the pediatric ward. The addition of observed patients to admissions leads to higher admission rates, yet does not reflect a true change in medical treatment. Even when OAs are combined with admissions there is still a clear decline in admission rates. Regardless of the categorization of OAs, there is a clear decline in both OAs and admissions over time. This reflects the process of clear decisions, aimed at lowering unnecessary hospital stay (OA or admission). In 2016–2017 it was decided to admit more of the OAs which led to a sharp decline in OAs compared to previous years.

An attempt to review the impact of the addition of the 16–17-year-old age group to national data was hampered by the fact that the health ministry’s data includes all of the 15–24-year old group as one. https://www.health.gov.il/PublicationsFiles/emergency_2017.pdf However, even with a limited ability to address the exact ages- we found a constant rate of both the referrals as well has return visits to the PED over the years 2011–2017 (years with available data).

In addition, two possible confounders to consider are readmission to another hospital as well as assessing the impact of the closure of a Jerusalem based general hospital (Bikur Cholim). Based on the available data from the health ministry there is a national level of return to other hospital of approximately 1.5% that has been constant for all hospitals over the past 10 years. Regarding the closing of a local hospital- we found that there was a constant decline in admission of pediatric patients until the final closure during 2012. There is a report of approximately 2000 children seen yearly at this hospital in the years 2011–2012. This number does not affect the overall rise during the years 2012–2017. A certain impact is possible yet likely not significant.

This study did not analyze the potential influence of relevant variables such as diagnoses (e.g. trauma vs. medical), time of arrival (daytime vs. nighttime, weekend/holidays vs. weekdays) and socioeconomic background, on admission and RV rates, subjects addressed in previous studies [14–16]. Although minor trauma cases were shifted to the PED from the adult ED, this was a gradual process only completed fully towards the end of 2017. We were unable to determine this subgroup’s information based on the hospital’s medical records.

In addition, an important factor that influences the adequacy of using RVs as a quality measure is the root causes of the RVs. These causes can affect the interpretation of the RVs in the context of quality. More studies on these causes are needed.

## Conclusion

This study demonstrates that the implementation of specific and focused measures in the PED can aid in the avoidance of unnecessary pediatric hospital admissions without leading to a higher RV rate. Such programs improve the quality of care given to children. Our study also supports the combined analysis of admission and RV rates as a more accurate indicator of the quality of care than the assessment of each one independently. Further multi-center studies are necessary to confirm these findings.

## Data Availability

All data generated or analysed during this study are included in this published article.

## Abbreviations

PED: Pediatric emergency department
SZMC: Shaare Zedek Medical Center
RV: Return visit
OA: Observational admission
QA: Quality assurance
